# Efficacy analysis of icaritin combined with transarterial chemoembolization in hepatocellular carcinoma stratified by different child-pugh classes

**DOI:** 10.3389/fimmu.2026.1876151

**Published:** 2026-07-30

**Authors:** Jingxuan Xu, Yao Xing, Fenhua Zhao, Bufu Tang, Xinghai Li, Jiangping Cun, Huilin Lu, Xiangwen Xia, Hongjie Fan, Yuting Gu, Tao Xu

**Affiliations:** 1 Department of Radiology, Dongyang Hospital Affiliated to Wenzhou Medical University, Dongyang, Zhejiang, China; 2 School of Clinical Medicine, North Sichuan Medical College, Nanchong, Sichuan, China; 3 Department of Radiation Oncology, Zhongshan Hospital, Fudan University, Shanghai, China; 4 Department of Minimally Invasive Intervention, Ganzhou People’s Hospital, Ganzhou, China; 5 Department of Radiology, the Second Affiliated Hospital of Kunming Medical University, Kunming, China; 6 Department of Interventional Therapy, Xinxiang Central Hospital/the Fourth Clinical College of Xinxiang Medical University, Xinxiang, Henan, China; 7 Department of Radiology, Union Hospital, Tongji Medical College, Huazhong University of Science and Technology, Wuhan, China; 8 Hubei Provincial Clinical Research Center for Precision Radiology & Interventional Medicine, Wuhan, China; 9 Hubei Provincial Key Laboratory of Molecular Imaging, Wuhan, China; 10 Department of Radiology, The First People’s Hospital of Yunnan Province, Kunming University of Science and Technology Affiliated Hospital, Kunming, China

**Keywords:** hepatocellular carcinoma, icaritin, immunomodulator, overall survival, transarterial chemoembolization

## Abstract

**Objective:**

To evaluate if transarterial chemoembolization (TACE) combined with Icaritin provides additional survival benefits compared to TACE alone in intermediate-to-advanced hepatocellular carcinoma (HCC) across different Child-Pugh classes.

**Background:**

TACE is a standard locoregional therapy for intermediate-to-advanced HCC, yet monotherapy yields limited long-term survival. Icaritin, an immunomodulator, demonstrates survival benefits in advanced HCC with a favorable safety profile lacking severe hepatotoxicity or bone marrow suppression.

**Methods:**

This multicenter retrospective cohort study included patients with Barcelona Clinic Liver Cancer (BCLC) stage B and C HCC. Propensity score matching (PSM) was utilized to balance baseline covariates between the TACE with Icaritin and TACE alone groups. The primary endpoint was overall survival (OS).

**Results:**

Following PSM, 250 patients were evaluated. In the Child-Pugh grade A group, TACE combined with Icaritin was associated with longer median OS than TACE alone (28.8 vs. 15.7 months; HR, 0.42; 95% CI, 0.29-0.62; P<0.001). Median PFS was also longer in the combination group (9.7 vs. 8.1 months; HR, 0.66; 95% CI, 0.49-0.90; P = 0.007). In Child-Pugh subgroup analyses, the treatment effect was most consistent in patients with Child-Pugh grade A liver function. In Child-Pugh grade B patients, OS favored the combination therapy, whereas the PFS result was not statistically definitive. ORR (49.6% vs. 47.2%) and DCR (83.2% vs. 74.4%) were comparable. The combination regimen did not significantly increase grade 3–4 liver function-related adverse events.

**Conclusions:**

TACE combined with Icaritin was associated with improved survival outcomes, with the most consistent benefit observed in patients with Child-Pugh grade A liver function. In Child-Pugh grade B patients, the findings should be interpreted cautiously because of the limited subgroup size and insufficient statistical power.

## Introduction

Hepatocellular carcinoma (HCC) has a high incidence and mortality rate worldwide and is the third leading cause of cancer-related deaths (1, 2). Most patients with HCC are diagnosed at intermediate to advanced stages, and transarterial chemoembolization (TACE) is a standard locoregional treatment modality. However, monotherapy has significant limitations, often leading to tumor residue, increased risk of recurrence, and limited long-term survival benefits. Moreover, vulnerable patients with impaired liver function are frequently excluded from large randomized controlled trials. When receiving conventional high-intensity systemic therapy, they face a high risk of severe adverse events (AEs), liver failure, and treatment discontinuation, making it difficult to achieve a balance between efficacy and safety (3). Therefore, exploring a treatment regimen that can both effectively control the tumor and accommodate patients with fragile liver function is a key clinical challenge that urgently needs to be addressed.

Icaritin, a small-molecule immunomodulator extracted from the traditional Chinese herb Epimedium, has shown unique potential in the treatment of advanced HCC. A randomized, double-blind phase III trial (SNG1705 ICR-1) demonstrated that Icaritin significantly prolonged overall survival (OS) in patients with advanced HCC who had specific biomarkers (CBS-positive), with a favorable safety profile (4). Studies have shown that Icaritin reduces the generation and activation of myeloid-derived suppressor cells (MDSCs) by downregulating tumor-associated extramedullary hematopoiesis (EMH) and inhibiting signal transducer and activator of transcription 3 (STAT3) activity, thereby enhancing anti-tumor immunity and inhibiting HCC proliferation (5, 6). Furthermore, its immunomodulatory effects can induce mitochondrial apoptosis, trigger immunogenic cell death of tumor cells, and remodel the tumor microenvironment (7). Additionally, Icaritin mediates the inhibition of alpha-fetoprotein (AFP) transcription by upregulating p53 protein expression. This effect is achieved through direct interaction of p53 with the p53 DNA-binding element in the AFP gene promoter region and by regulating chromatin structure remodeling in the core promoter region, ultimately significantly inhibiting AFP synthesis and secretion (8, 9). The core advantage of Icaritin lies in its lack of severe vascular endothelial damage and bone marrow suppression, offering a safer treatment option for patients with fragile liver function.

Based on the above background, we conducted this multicenter retrospective cohort study to evaluate whether TACE combined with Icaritin provides additional survival benefits compared to TACE alone in patients with intermediate to advanced HCC. In particular, this study focused on analyzing the differences in clinical efficacy and safety of this combination regimen across different baseline liver function statuses (Child-Pugh grade A vs. B), aiming to provide more comprehensive and robust evidence for the clinical application of this novel immunomodulator-based combination therapy in real-world high-risk and vulnerable populations.

## Materials and methods

### Study design and patient population

**Figure 1:**
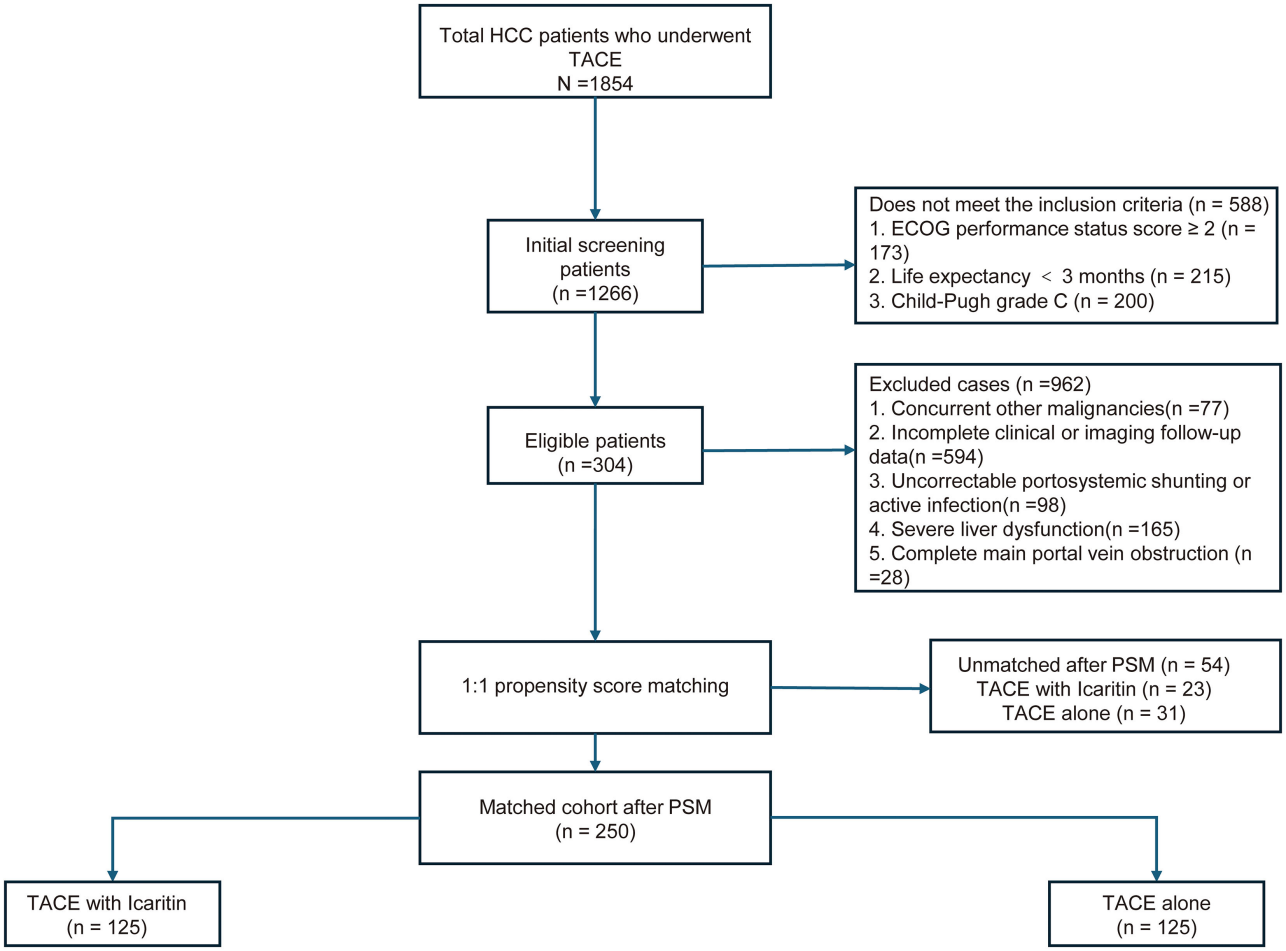
Flowchart of patient selection and propensity score matching.

This study was a multicenter, retrospective cohort study with data collected from August 2022 to November 2025, covering four regional medical centers in China. The study protocol was approved by the ethics review committees of each center (IRB No. IEC 2024 1130), and all clinical data collection and processing strictly followed the ethical standards of the Declaration of Helsinki. The study primarily included patients diagnosed with Barcelona Clinic Liver Cancer (BCLC) stage B and C HCC. To address potential heterogeneity between BCLC stage B and stage C disease, BCLC stage C was defined as ECOG performance status 1–2 and/or the presence of portal vein tumor thrombus or extrahepatic metastasis; patients without these features were classified as BCLC stage B. Considering the large number of patients with underlying liver disease in the real world, the inclusion criteria not only included patients with well-compensated liver function (Child-Pugh grade A) but also included some patients with impaired liver function (Child-Pugh grade B). Other inclusion criteria were: Eastern Cooperative Oncology Group (ECOG) performance status score of 0 or 1; some patients with ECOG score 2 who met the strict criteria for TACE and could tolerate the procedure; life expectancy >3 months; and presence of portal vein tumor thrombosis (PVTT) that was limited without complete obstruction of the main portal vein. Exclusion criteria included: concurrent other malignancies; complete main portal vein obstruction unsuitable for embolization; severe coagulation disorders, hepatic encephalopathy, refractory ascites, or recent history of esophagogastric variceal bleeding; uncorrectable portosystemic shunting or active infection; and incomplete clinical or imaging follow-up data (**Figure 1**).

### TACE procedure

All patients underwent a comprehensive baseline assessment before the initial treatment, including routine blood tests, liver and kidney function tests, coagulation parameters, and contrast-enhanced abdominal CT or MRI to determine tumor burden, vascular anatomy, and portal vein patency. TACE procedures were performed by interventional radiologists with over 8 years of experience according to the “Clinical Application Management Standards and Quality Control Indicators for TACE in the Treatment of Liver Cancer (2024 Edition)”. The procedure was performed under local anesthesia using the Seldinger technique via femoral artery puncture. A 5Fr vascular sheath and a Yashiro catheter were used to locate the celiac trunk and superior mesenteric artery. A 2.7 Fr Progreat^®^ microcatheter was used for superselective catheterization into the target artery supplying the tumor (subsegmental or more distal branches). Subsequently, a chemotherapeutic emulsion (doxorubicin or cisplatin mixed with lipiodol) or drug-eluting beads was slowly injected until blood flow nearly stagnated. Finally, gelatin sponge particles were used for supplementary embolization to ensure complete blockage of tumor blood supply. After the procedure, the catheter was removed, compression hemostasis was applied at the puncture site, and symptomatic supportive care including hydration, analgesia, and antiemetics was provided.

### Systemic treatment regimen

In the combination therapy group, patients started oral Icaritin after TACE, at a standard dose of 600 mg twice daily, taken after meals. During treatment, clinicians closely monitored changes in patients’ liver function. If drug-related liver function abnormalities occurred, temporary dose interruption or reduction was allowed, and treatment was resumed after symptom relief. During follow-up, if clinically indicated, the addition of other targeted agents (e.g., lenvatinib, donafenib, or sorafenib) was permitted according to clinical guidelines.

### Follow-up and efficacy assessment

Patients were followed 4–6 weeks after initial TACE with contrast-enhanced CT or MRI, and tumor response was assessed using the modified Response Evaluation Criteria in Solid Tumors (mRECIST) (10). Repeat TACE was performed on demand for residual viable tumors if liver function permitted. AFP was monitored every 1–2 months, and imaging was repeated every 3 months. The primary endpoint of this study was OS, defined as the time from the date of initial TACE to death from any cause or last follow-up. The secondary endpoint was progression-free survival (PFS), defined as the time from treatment initiation to the first imaging-confirmed disease progression or death (11). Additionally, objective response rate (ORR, defined as the sum of complete response and partial response) and disease control rate (DCR, defined as the sum of complete response, partial response, and stable disease) were calculated. All AEs were recorded and graded according to the National Cancer Institute Common Terminology Criteria for Adverse Events (CTCAE) version 4.03.

### Statistical analysis

Data were analyzed using SPSS 27.0 and R 4.5.1. Group comparisons utilized t-tests, Mann-Whitney U tests, chi-square, or Fisher’s exact tests as appropriate. Propensity score matching (PSM) and inverse probability of treatment weighting (IPTW) were applied to balance baseline covariates and minimize confounding. Survival was estimated using the Kaplan-Meier method and log-rank test. Multivariate Cox proportional hazards regression identified independent prognostic factors for OS and PFS, including variables with P < 0.05 from univariate analyses. The distribution of BCLC stage was compared between treatment groups before and after PSM. Additional BCLC-stratified Cox analyses and Cox models including BCLC stage as an adjustment factor were performed as sensitivity analyses. Subgroups included sex, age, ECOG performance status, Child-Pugh classification, ALBI grade, PVTT, extrahepatic metastasis, vascular invasion, AFP level, number of lesions, maximum tumor diameter, and sessions of TACE >=2. Cox models were used to estimate treatment effects within each subgroup, and treatment-by-subgroup interaction terms were tested. To account for potential delayed immune responses, landmark analyses evaluated long-term survival for OS (at 4, 6, and 8 months) and PFS (at 2, 4, and 6 months). Additionally, a competing risk model (Fine-Gray test) assessed the cumulative incidence of disease progression, accounting for non-tumor-specific mortality. Statistical significance was defined as a two-sided P < 0.05.

## Results

### Patients

Before PSM, a total of 304 patients were included in this study, with a median age of 55 years (range 25-80), and 90.5% had a history of hepatitis B virus infection. Before PSM, the median AFP value was 130.9 (IQR, 992.6) ng/ml; the mean maximum tumor diameter was 7.07 ± 3.84 cm. Furthermore, 52.7% of patients in the TACE with Icaritin group and 47.4% in the TACE alone group received two or more TACE sessions. Before PSM, BCLC stage B/C distributions were 53/103 (34.0%/66.0%) in the TACE-alone group and 64/84 (43.2%/56.8%) in the TACE with Icaritin group (P = 0.123). After PSM, the corresponding distributions were 44/81 (35.2%/64.8%) and 55/70 (44.0%/56.0%), respectively (P = 0.196), indicating no statistically significant imbalance by BCLC stage (**Supplementary Table 1**). To control baseline differences between the two groups, PSM was performed, resulting in 250 patients with balanced baseline characteristics (All P > 0.05) (**Table 1**; **Supplementary Table S2**; **Supplementary Figure 1**). Some patients experienced temporary treatment interruption, mainly due to treatment-related adverse events, additional anti-tumor interventions, additional TACE sessions, and combined targeted therapy. These interruptions lasted approximately 2 weeks to 2 months, after which all patients resumed the original treatment regimen. Dose reduction (Icaritin reduced to 200 mg once daily) was implemented in 8.0% of patients, who subsequently completed treatment. No patient permanently discontinued the TACE with Icaritin regimen due to serious adverse events.

**Table 1:** Comparison of baseline characteristics for group between TACE with icaritin vs TACE alone after matching.

Characteristics	Unmatched			Matched		
	TACE alone n = 156 ^a^	TACE with Icaritin n = 148 ^a^	P ^b^	TACE alone n = 125 ^a^	TACE with Icaritin n = 125 ^a^	P ^b^
Sex			0.731			0.679
Male	141 (90%)	132 (89%)		113 (90%)	111 (89%)	
Female	15 (10%)	16 (11%)		12 (10%)	14 (11%)	
Age			0.826			0.698
<60	94 (60%)	91 (61%)		74 (59%)	77 (62%)	
≥ 60	62 (40%)	57 (39%)		51 (41%)	48 (38%)	
ECOG ^c^			0.926			>0.999
0	104 (67%)	101 (68%)		84 (67%)	85 (68%)	
1	50 (32%)	45 (30%)		39 (31%)	38 (30%)	
2	2 (1%)	2 (1%)		2 (2%)	2 (2%)	
Targeted therapy			0.814			0.961
None	49 (31%)	46 (31%)		38 (30%)	36 (29%)	
Lenvatinib	59 (38%)	60 (41%)		51 (41%)	54 (43%)	
Donafenib	38 (24%)	36 (24%)		31 (25%)	29 (23%)	
Regorafenib	10 (6%)	6 (4%)		5 (4%)	6 (5%)	
Extrahepatic metastases^d^			0.787			0.625
No	142 (91%)	136 (92%)		115 (92%)	117 (94%)	
Yes	14 (9%)	12 (8%)		10 (8%)	8 (6%)	
Viral infection			0.156			0.811
No	2 (1%)	8 (5%)		2 (2%)	2 (2%)	
Hepatitis B	142 (91%)	133 (90%)		120 (96%)	117 (94%)	
Hepatitis C	9 (6%)	6 (4%)		3 (2%)	5 (4%)	
Other	3 (2%)	1 (1%)		0 (0%)	1 (1%)	
Portal vein tumor thrombus			0.062			0.998
None	73 (47%)	92 (62%)		68 (54%)	70 (56%)	
Type I	24 (15%)	20 (14%)		20 (16%)	20 (16%)	
Type II	49 (31%)	29 (20%)		31 (25%)	29 (23%)	
Type III	9 (6%)	6 (4%)		5 (4%)	5 (4%)	
Type IV	1 (1%)	1 (1%)		1 (1%)	1 (1%)	
AFP≥400 ng/mL			0.349			0.507
No	106 (68%)	93 (63%)		84 (67%)	79 (63%)	
Yes	50 (32%)	55 (37%)		41 (33%)	46 (37%)	
Number of lesions			0.115			0.471
≤ 3	121 (78%)	103 (70%)		95 (76%)	90 (72%)	
>3	35 (22%)	45 (30%)		30 (24%)	35 (28%)	
Maximum diameter of lesion			0.181			0.296
<5 cm	61 (39%)	47 (32%)		51 (41%)	43 (34%)	
≥ 5 cm	95 (61%)	101 (68%)		74 (59%)	82 (66%)	
Vascular invasion			0.002			0.722
No	3 (2%)	15 (10%)		3 (2%)	5 (4%)	
Yes	153 (98%)	133 (90%)		122 (98%)	120 (96%)	

TACE, transarterial chemoembolization; ECOG, Eastern Cooperative Oncology Group; AFP, alpha-Fetoprotein. ^a^n (%). ^b^ Pearson’s Chi-squared test; Wilcoxon rank sum test; Fisher’s exact test. ^c^ ECOG score of 0 indicates that patients are fully active and able to carry on all pre-disease activities without restriction, and 1 indicates that patients are restricted in physically strenuous activity but is ambulatory and able to carry out work of a light nature, including self-care. d Extrahepatic metastases include metastasis to one or more sites such as the lung, bone, and peritoneum.

### OS and PFS

**Figure 2:**
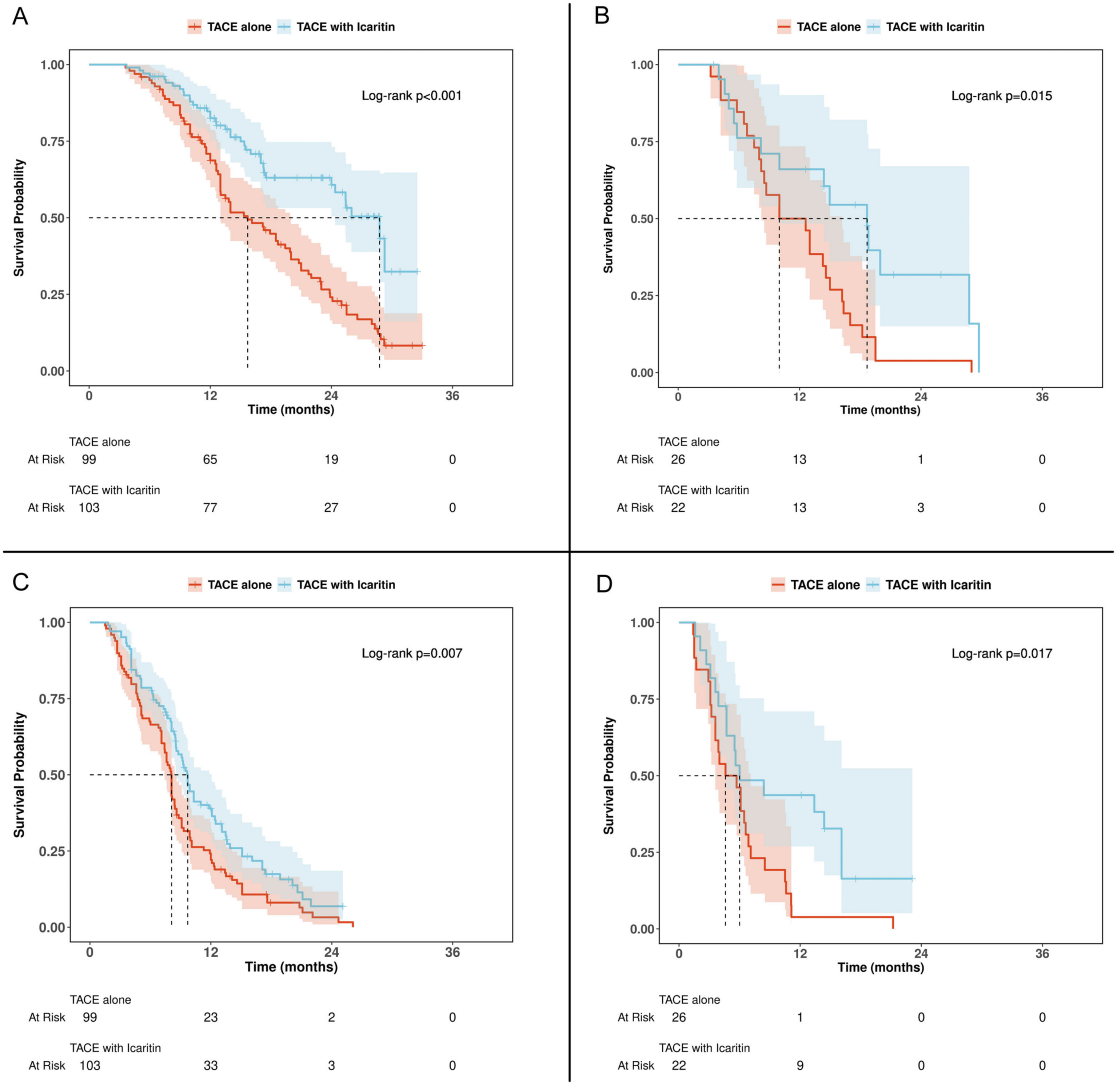
Kaplan-Meier curves of overall survival and progression-free survival in two groups under different child-pugh grades (A and B). **(A)** Overall survival periods of the two groups under Child-Pugh grade A. **(B)** Overall survival periods of the two groups under Child-Pugh grade B. **(C)** Progression-free survival in both groups under Child-Pugh grade A. **(D)** Progression-free survival in both groups under Child-Pugh grade B.

Before matching, the median follow-up duration was 14.2 months (range 3.2-33.0), and the overall number of deaths was 253 (83.2%). Kaplan-Meier survival analysis showed that, across different Child-Pugh grades, TACE with Icaritin significantly improved patient survival outcomes compared to TACE alone: in Child-Pugh grade A patients, the median OS, 1-year OS rate, median PFS, and 1-year PFS rate were significantly better in the TACE with Icaritin group than in the TACE alone group (all P < 0.05); in Child-Pugh grade B patients, the median OS was significantly prolonged in the TACE with Icaritin group (P = 0.030) (**Supplementary Table 3**; **Figure 2**).

**Figure 3:**
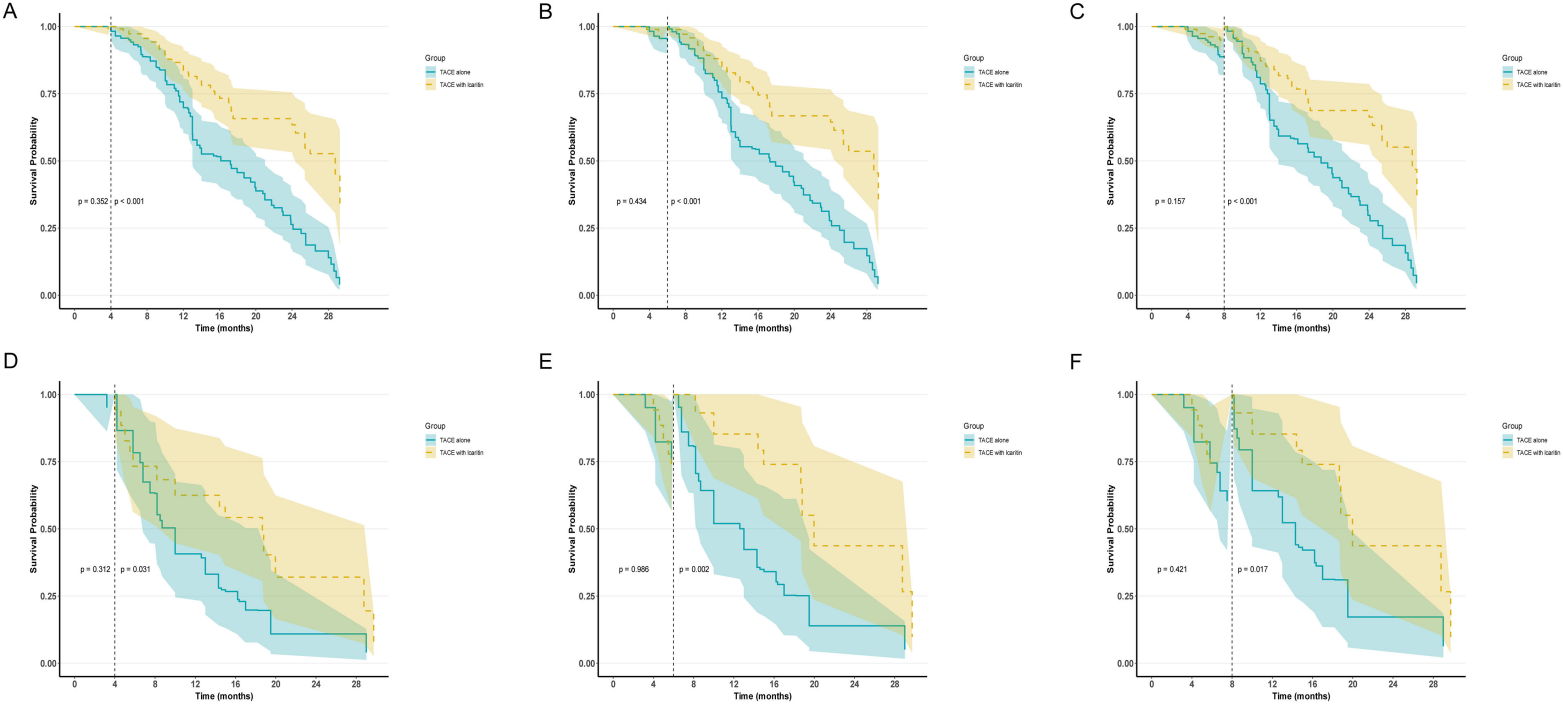
Landmark analysis of the overall survival of the two groups with the cut-off values of the 4th, 6th, and 8th months under different child-pugh grades. **(A)** Child-Pugh grade A, the cut-off value is the 4th month. **(B)** Child-Pugh grade A, the cut-off value is the 6th month. **(C)** Child-Pugh grade A, the cut-off value is the 8th month. **(D)** Child-Pugh grade B, the cut-off value is the 4th month. **(E)** Child-Pugh grade B, the cut-off value is the 6th month. **(F)** Child-Pugh grade B, the cut-off value is the 8th month.

**Figure 4:**
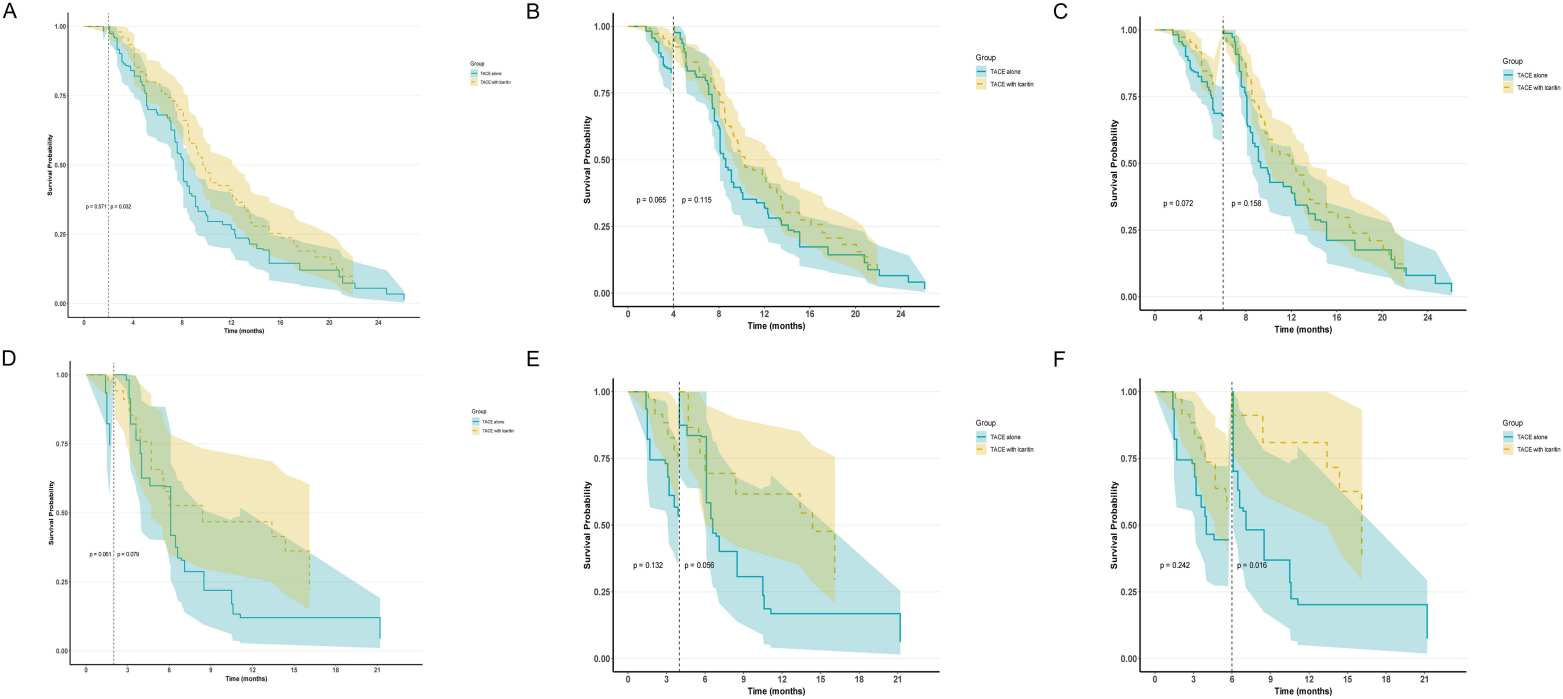
Landmark analysis of the progression-free survival of the two groups with the cut-off values of the 2nd, 4th, and 6th months under different child-pugh grades. **(A)** Child-Pugh grade A, the cut-off value is the 2nd month. **(B)** Child-Pugh grade A, the cut-off value is the 4th month. **(C)** Child-Pugh grade A, the cut-off value is the 6th month. **(D)** Child-Pugh grade B, the cut-off value is the 2nd month. **(E)** Child-Pugh grade B, the cut-off value is the 4th month. **(F)** Child-Pugh grade B, the cut-off value is the 6th month.

After matching, the median follow-up duration was 14.3 months, and the mortality rates in the TACE with Icaritin and TACE alone groups were 41.6% and 83.2%, respectively (P < 0.001). Multivariate Cox regression analysis confirmed that ECOG score 2, Child-Pugh grade B, portal vein tumor thrombosis, number of lesions ≥3, and vascular invasion were independent risk factors for OS (all P < 0.05) (**Table 2**); hepatitis B/C infection and portal vein tumor thrombosis were independent risk factors for PFS (all P < 0.05) (**Table 3**). In BCLC-stratified analyses of the matched cohort, TACE with Icaritin was associated with improved OS in both BCLC stage B (HR, 0.30; 95% CI, 0.15-0.59; P < 0.001) and BCLC stage C patients (HR, 0.53; 95% CI, 0.36-0.78; P = 0.001). For PFS, the association favored the combination group in BCLC stage B (HR, 0.72; 95% CI, 0.47-1.11; P = 0.133) and was significant in BCLC stage C (HR, 0.58; 95% CI, 0.41-0.82; P = 0.002). In additional Cox models including BCLC stage as an adjustment factor, TACE with Icaritin remained independently associated with better OS (HR, 0.50; 95% CI, 0.35-0.70; P < 0.001) and PFS (HR, 0.62; 95% CI, 0.47-0.81; P < 0.001), while BCLC stage C was associated with worse OS and PFS (**Supplementary Table 4**). Landmark and Kaplan-Meier survival analyses showed that, across different Child-Pugh grades, overall survival was significantly better in the TACE with Icaritin group than in the TACE alone group at all key time points (**Figure 3**). Among patients surviving beyond 4 months, the median OS in the two groups was 29.1 (vs. 16.7) months and 18.5 (vs. 8.8) months in the Child-Pugh grade A/B subgroups, respectively (Both P < 0.05). Similar survival differences were observed at 6 and 8 months. For PFS, differences between the two groups were only statistically significant in the Child-Pugh grade A subgroup at 2 months and in the Child-Pugh grade B subgroup at 6 months (P = 0.032 and P = 0.016) (**Figure 4**). In landmark analyses, treatment differences were not significant before the landmark event but became significant thereafter.

**Table 2:** Univariable and multivariable cox regression analysis of factors associated with overall survival in HCC patients.

Characteristic	Univariable			Multivariable		
	HR	95%CI	P	HR	95%CI	P
Sex						
Male						
Female	0.49	0.29, 0.84	0.010	0.40	0.23, 0.68	<0.001
Age						
<60 yrs						
≥ 60 yrs	1.13	0.83, 1.55	0.440			
ECOG						
0						
1	2.93	2.10, 4.07	<0.001	2.00	1.43, 2.79	<0.001
2	8.71	2.68, 28.25	<0.001	3.38	1.05, 10.83	0.041
Child-Pugh classification						
Grade A						
Grade B	2.10	1.46, 3.02	<0.001	1.33	0.92, 1.91	0.046
Targeted therapy						
None						
Lenvatinib	1.13	0.76, 1.68	0.547	1.01	0.74, 1.39	0.933
Donafenib	1.23	0.79, 1.93	0.362	1.34	0.93, 1.94	0.119
Regorafenib	2.12	1.10, 4.08	0.024	1.65	0.91, 3.00	0.099
Sessions of TACE ≥2						
No						
Yes	0.98	0.72, 1.35	0.919			
Extrahepatic metastases						
No						
Yes	2.54	1.43, 4.50	0.001	1.16	0.65, 2.06	0.616
Viral infection						
None						
Hepatitis B	0.01	0.00, 0.03	<0.001	0.02	0.01, 0.05	<0.001
Hepatitis C	0.01	0.00, 0.05	<0.001	0.03	0.01, 0.07	<0.001
Other	0.04	0.00, 0.53	0.014	0.09	0.01, 0.67	0.018
Portal vein tumor thrombus						
None						
Type I	2.98	1.89, 4.71	<0.001	2.88	1.91, 4.34	<0.001
Type II	4.36	2.97, 6.40	<0.001	3.16	2.26, 4.43	<0.001
Type III	9.84	5.12, 18.93	<0.001	8.50	4.51, 16.03	<0.001
Type IV	NA	NA	NA	NA	NA	NA
AFP ≥400 ng/mL						
No						
Yes	1.17	0.86, 1.61	0.322			
Number of lesions						
≤ 3						
>3	2.71	1.75, 4.20	<0.001	1.89	1.22, 2.93	0.004
Maximum diameter of lesion						
<5 cm						
≥ 5 cm	1.48	1.02, 2.13	0.037	1.03	0.71, 1.50	0.863
Vascular invasion						
No						
Yes	2.81	1.24, 6.35	0.013	2.42	1.07, 5.49	0.034

ECOG, Eastern Cooperative Oncology Group performance status; HR, hazard ratio; CI, confidence interval; AFP, alpha-fetoprotein; Inf, infinity. Variables with P < 0.1 in univariate analysis were entered into the multivariate Cox model, which adjusted for all listed covariates. Reference groups: “none” for viral infection and PVTT type. The HR for PVTT type IV was unstable due to small sample size and should be interpreted with caution. HR, 95% CI, and P value for PVTT Type IV were not reported because the estimate was unstable due to sparse data in this subgroup.

**Table 3:** Univariable and multivariable cox regression analysis of factors associated with progression-free survival in HCC patients.

Characteristic	Univariable			Multivariable		
	HR	95%CI	P	HR	95%CI	P
Sex						
Male						
Female	0.70	0.47, 1.06	0.093	0.70	0.45, 1.08	0.106
Age						
<60 yrs						
≥ 60 yrs	0.99	0.76, 1.29	0.936			
ECOG						
0						
1	1.51	1.12, 2.03	0.007	1.23	0.88, 1.72	0.230
2	3.18	1.01, 10.04	0.049	2.16	0.61, 7.68	0.235
Child-Pugh classification						
Grade A						
Grade B	1.40	1.00, 1.96	0.052	1.17	0.80, 1.72	0.405
Targeted therapy						
None						
Lenvatinib	1.14	0.82, 1.59	0.435			
Donafenib	1.33	0.91, 1.93	0.140			
Regorafenib	1.59	0.85, 2.98	0.147			
Sessions of TACE ≥2						
No						
Yes	1.11	0.85, 1.46	0.430			
Extrahepatic metastases						
No						
Yes	1.63	0.96, 2.78	0.071	1.32	0.74, 2.34	0.350
Viral infection						
None						
Hepatitis B	0.01	0.00, 0.05	<0.001	0.02	0.01, 0.10	<0.001
Hepatitis C	0.02	0.00, 0.08	<0.001	0.03	0.01, 0.15	<0.001
Other	0.09	0.01, 1.01	0.050	0.15	0.01, 1.82	0.137
Portal vein tumor thrombus						
None						
Type I	1.81	1.23, 2.66	0.003	1.65	1.10, 2.46	0.015
Type II	1.68	1.22, 2.33	0.002	1.42	1.01, 2.00	0.044
Type III	2.65	1.45, 4.86	0.002	1.82	0.89, 3.71	0.098
Type IV	2.51	0.35, 18.12	0.361	3.31	0.44, 25.01	0.246
AFP ≥400 ng/mL						
No						
Yes	0.95	0.72, 1.25	0.716			
Number of lesions						
≤ 3						
>3	1.51	1.10, 2.07	0.012	1.27	0.91, 1.79	0.165
Maximum diameter of lesion						
<5 cm						
≥ 5 cm	1.30	0.97, 1.75	0.078	1.13	0.83, 1.55	0.432
Vascular invasion						
No						
Yes	0.98	0.63, 1.55	0.947			

ECOG, Eastern Cooperative Oncology Group performance status; HR, hazard ratio; CI, confidence interval; AFP, alpha-fetoprotein. Variables with P < 0.1 in univariate analysis were entered into the multivariate Cox model, which adjusted for sex, ECOG score, Child-Pugh class, extrahepatic metastases, viral infection, portal vein tumor thrombus (PVTT) type, number of lesions, and maximum diameter of lesion. Reference groups: “none” for both viral infection and PVTT type. Variables with P≥ 0.1 in univariate analysis (age, targeted therapy, AFP ≥400 ng/mL, vascular invasion) were excluded.

To further examine whether the survival association was independent of early radiological response, we performed response-adjusted Cox sensitivity analyses in the matched cohort. After adjustment for best mRECIST response category, additional targeted therapy during follow-up, and key baseline prognostic factors, TACE with Icaritin remained independently associated with improved OS (HR, 0.49; 95% CI, 0.35–0.70; P < 0.001) and PFS (HR, 0.60; 95% CI, 0.45–0.81; P < 0.001). The full univariable and multivariable models are presented in **Supplementary Tables 5**, **6**.

The subgroup forest plots after PSM are shown in **Supplementary Figures 2** and **S3**. Overall, the HRs in most subgroups favored TACE with Icaritin. The survival benefit was observed in both Child-Pugh grade A and grade B patients, with no significant interaction by Child-Pugh classification for OS (P for interaction=0.950) or PFS (P for interaction=0.114). For OS, a significant interaction was observed for PVTT status (P for interaction=0.012), with a stronger benefit in patients without PVTT (HR, 0.31; 95% CI, 0.18-0.54) than in those with PVTT (HR, 0.76; 95% CI, 0.51-1.15). For PFS, the interaction by ALBI grade was significant (P for interaction=0.023), although the ALBI grade 3 subgroup was small. Patients receiving two or more TACE sessions also showed a more pronounced association with improved OS and PFS (Both P < 0.05).

### Sensitivity analysis

**Figure 5:**
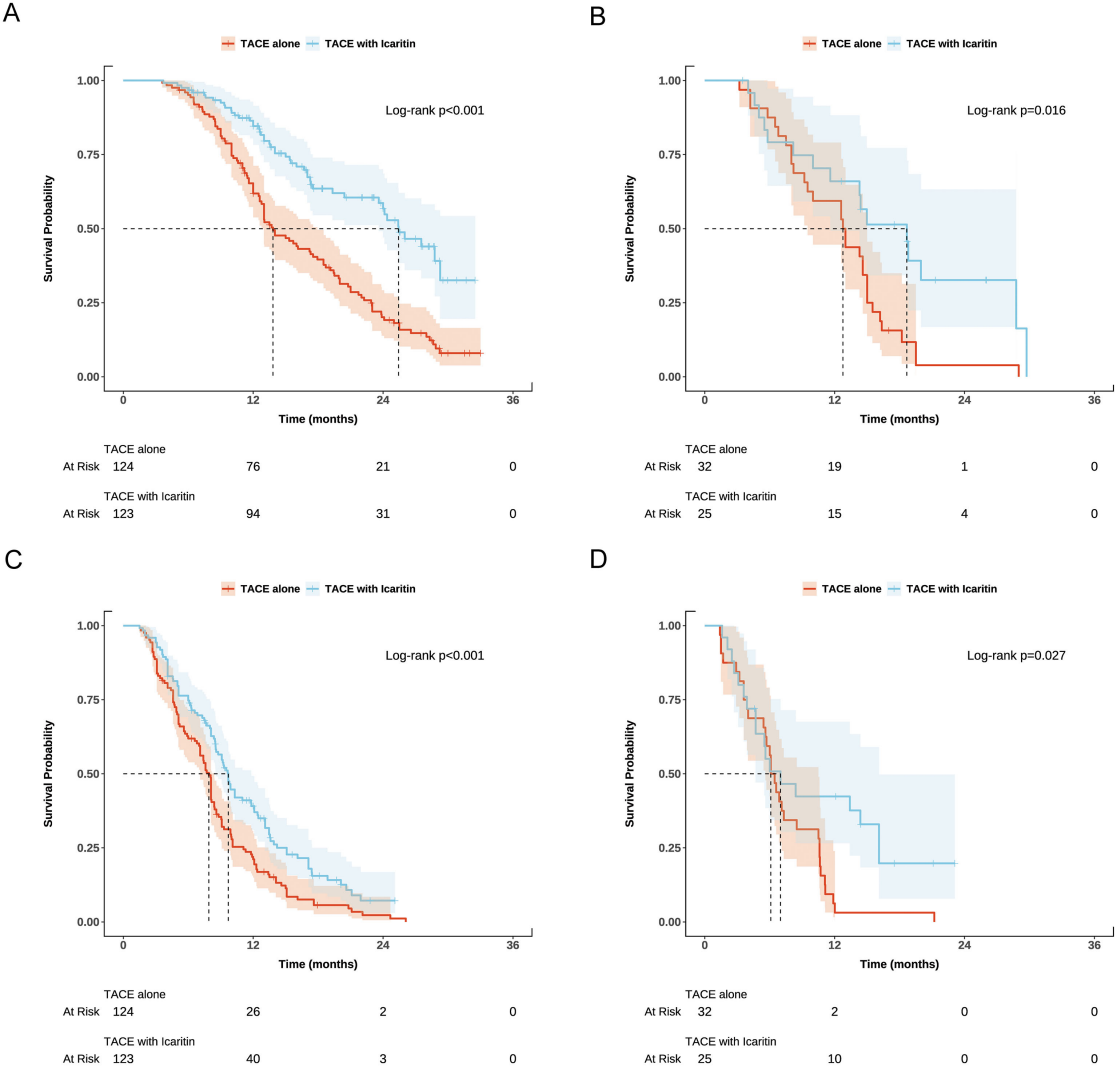
Kaplan-Meier curves of overall survival and progression-free survival in two groups under different child-pugh grades (A and B) after IPTW. **(A)** Overall survival periods of the two groups under Child-Pugh grade A. **(B)** Overall survival periods of the two groups under Child-Pugh grade B. **(C)** Progression-free survival in both groups under Child-Pugh grade A. **(D)** Progression-free survival in both groups under Child-Pugh grade B. IPTW, inverse probability of treatment weighting.

IPTW sensitivity analysis based on propensity scores was performed. After weighting, baseline characteristics were well balanced between the treatment groups (most standardized mean differences < 0.1) (**Supplementary Figure 4**). In the IPTW-weighted sensitivity analysis, both Child-Pugh grade A and Child-Pugh grade B subgroups were evaluated separately. Among patients with Child-Pugh grade A liver function, TACE with Icaritin was associated with longer OS than TACE alone, with a median OS of 25.4 months (95% CI, 20.6-not estimable) versus 15.0 months (95% CI, 13.0-19.1), respectively (weighted Cox HR, 0.46; 95% CI, 0.33-0.65; P < 0.001). A consistent survival benefit was also observed in the Child-Pugh grade B subgroup, although with wider confidence intervals because of the smaller sample size: median OS was 15.0 months (95% CI, 14.4-not estimable) with TACE with Icaritin versus 12.8 months (95% CI, 8.2-15.5) with TACE alone (weighted Cox HR, 0.48; 95% CI, 0.25-0.94; P = 0.031). The weighted 12- and 24-month OS rates were 83.7% and 34.4% versus 64.5% and 22.7% in Child-Pugh grade A patients, and 70.0% and 21.1% versus 56.4% and 4.7% in Child-Pugh grade B patients, respectively (**Supplementary Table 7**). A similar improvement trend was observed for progression-free survival (**Figure 5**). Weighted Kaplan-Meier curves and landmark analyses also showed a clear survival benefit for the combination therapy group (**Supplementary Figures 5**-**S7**). These results were consistent with those from the propensity score-matched cohort. Competing risk analysis (event = disease progression; competing event = death) showed that in Child-Pugh grade A patients, the cumulative incidence of progression at 10 months was 54.4% (95% CI 43.8-62.8%) in the TACE with Icaritin group, compared to 70.3% (95% CI 61.9-80.3%) in the TACE alone group (Gray’s test P = 0.002)(**Supplementary Figure 8**). There was no significant difference in the cumulative incidence of death between the two groups, suggesting that the observed difference in disease progression was unlikely driven by competing mortality.

### Tumor response

Based on mRECIST criteria, there were no statistically significant differences between the TACE combined with Icaritin group and the TACE monotherapy group in complete response, partial response, stable disease, and progressive disease (P > 0.05). Regarding the best tumor response after treatment, over 70% of patients in both groups responded well with tumor control, while 15% of patients showed tumor enlargement or new lesions after treatment. In the TACE alone group, the objective response rate was 47.2%, the disease control rate was 74.4%, and the progressive disease rate was 26.4%; in the TACE combined with Icaritin group, the objective response rate was 49.6%, the disease control rate was 83.2%, and the progressive disease rate was 16.8%. There were no statistically significant differences in objective response rate or disease control rate between the two groups (P > 0.05) (**Table 4**).

**Table 4:** Best overall tumor response before and after propensity score matching.

Variables	Before matching			After matching		
	TACE alone n=156^a^	TACE with Icaritin n=148^a^	P	TACE alone n=125^a^	TACE With Icaritin n=125^a^	P^b^
CR(%)	11 (7.1)	17 (11.5)	0.181	7 (5.6)	12 (9.6)	0.233
PR (%)	63 (40.4)	58 (39.2)	0.831	52 (41.6)	50 (40.0)	0.797
SD(%)	39 (25.0)	47 (31.8)	0.191	33 (26.4)	42 (33.6)	0.214
PD(%)	43 (27.6)	26 (17.6)	0.038	33 (26.4)	21 (16.8)	0.065
ORR(%)	74 (47.4)	75 (50.7)	0.572	59 (47.2)	62 (49.6)	0.704
DCR(%)	114 (73.1)	122 (82.4)	0.050	93 (74.4)	104 (83.2)	0.089

CR, Complete Response; PR, Partial Response; SD, Stable Disease; PD, Progressive Disease; ORR, Objective Response Rate; DCR, Disease Control Rate; ^a^(%). ^b^Pearson’s Chi-squared test; Wilcoxon rank sum test; Fisher’s exact test. ^c^ Objective response rate (ORR) was defined as the proportion of patients achieving complete response (CR) or partial response (PR), and disease control rate (DCR) was defined as the proportion of patients achieving CR, PR, or stable disease (SD).

### Adverse events

The main manifestations in both groups were post-embolization liver injury-related reactions, including dull pain in the liver area and transient elevations in serum total bilirubin and liver enzymes (ALT, AST, GGT), with no statistically significant differences in incidence between the two groups (P > 0.05). The incidences of mild-to-moderate ascites worsening and hypoalbuminemia did not differ significantly between groups. However, these events were concentrated in patients with Child-Pugh grade B and improved after symptomatic treatments such as hepatoprotective therapy and albumin supplementation, with no cases of severe liver failure. There were no statistically significant differences in the overall incidence of grade 3–4 liver function-related AEs and serious adverse events between the two groups (P > 0.05), suggesting that the combination regimen did not significantly increase the risk of severe hepatotoxicity in HCC patients (**Supplementary Table 8**). In this study, treatment adjustments were made in 21 patients (8.4%), of which 11 (4.4%) had treatment interrupted due to liver function fluctuations, and 10 (4.0%) had Icaritin dose reduction. No patient required permanent treatment discontinuation due to drug intolerance.

## Discussion

This study analyzed data from multicenter HCC patients, focusing on differences in survival benefits between TACE combined with Icaritin and TACE alone across different Child-Pugh grades. The results showed that patients in the TACE with Icaritin group had longer median OS and PFS, particularly with a significantly improved 1-year survival rate. An innovative aspect of this study is that it is the first to evaluate the efficacy of combining Icaritin with TACE in patients with different liver function statuses and to explore whether the association between TACE plus Icaritin and survival differed according to baseline liver function. The apparent dissociation between similar ORR/DCR and improved long-term survival warrants careful interpretation. First, **Table 4** does not show worse tumor control with the combination regimen: PR was slightly lower, but CR, SD, DCR, and the proportion without PD were numerically more favorable after matching. Second, ORR and DCR are categorical snapshots of radiologic response, whereas OS and PFS integrate response durability, later intrahepatic and extrahepatic progression, liver functional reserve, treatment tolerability, and subsequent management. In TACE-treated HCC, sustained response duration has been shown to correlate more strongly with OS than initial or best response alone (12). In systemic therapy trials for advanced HCC, objective response by mRECIST is prognostic, but its trial-level surrogacy for OS is only moderate, meaning survival benefit may not be fully explained by ORR differences alone (13). Thus, a survival advantage without a large ORR increment is biologically plausible if the regimen prolongs disease stabilization, delays progression, or preserves the ability to continue treatment.

International guidelines consistently recommend TACE as standard treatment for patients with BCLC stage B (equivalent to CNLC stage IIa/IIb), but patients with impaired liver function (e.g., Child-Pugh B) derive limited benefit (14). However, repeated TACE procedures can easily lead to destruction of the tumor’s feeding vascular bed and exacerbation of hypoxia in the hepatic microenvironment, inducing overexpression of HIF-1α and VEGF. This allows residual tumor cells to undergo malignant clonal evolution, easily developing into TACE refractoriness. Furthermore, intensive interventional procedures continuously deplete the patient’s liver function reserve, leading to high recurrence rates and limited survival in BCLC stage B HCC (15, 16). In recent years, the advantages of “locoregional + systemic” combination therapy have been increasingly recognized and validated. A randomized phase III study showed that compared with TACE alone, TACE combined with sorafenib significantly prolonged OS (22.2 months vs. 15.1 months) and PFS (16.2 months vs. 11.8 months) (17). Our study applied Icaritin, a drug approved by China’s NMPA for advanced HCC, as a new strategy combined with TACE, and the observed survival benefits are consistent with the broader rationale for TACE-based combination therapy, but should not be interpreted as direct evidence equivalent to randomized trial data. Mo et al. found that Icaritin can bind to the MyD88/IKKα protein complex, block the IL-6/JAK/STAT3 signaling pathway, reduce the secretion of pro-inflammatory cytokines such as TNF-α and IL-6, and downregulate PD-L1 expression (18). This action can remodel the tumor immune microenvironment and restore tumor-specific T cell function. These mechanisms of Icaritin complement the inhibitory immune microenvironment induced by TACE, enhancing the applicability of the clinical strategy.

In the present cohort, baseline AFP level was not an independent prognostic factor for OS or PFS. This may be because the Cox models already included more direct indicators of tumor aggressiveness, such as portal vein tumor thrombus, vascular invasion, tumor burden, ECOG status, and liver function. In this setting, dichotomized AFP level may not provide additional prognostic information. This finding should not be interpreted as denying the clinical relevance of AFP, but rather as indicating that AFP was not independently associated with survival in the adjusted model.

AASLD and EASL guidelines typically recommend direct systemic therapy alone for patients with BCLC stage C (14, 19). However, clinical practice in Asia suggests that for patients with acceptable liver function and without complete main portal vein obstruction, TACE can rapidly reduce local tumor burden and control blood supply to the tumor thrombus, thereby providing a therapeutic window for subsequent systemic therapy (20, 21). The evidence base for Icaritin is different from that of established first-line systemic regimens. In the Chinese Guidelines for the Diagnosis and Treatment of Primary Liver Cancer (2022 Edition), Icaritin is listed under “Other Treatments/Traditional Chinese Medicine,” with evidence grade 2 and recommendation B, rather than in the main systemic therapy section where agents such as sorafenib, lenvatinib, and atezolizumab plus bevacizumab are discussed at a higher evidence tier (22). Its regulatory evidence also comes mainly from biomarker-selected monotherapy studies, particularly CBS-positive or peripheral blood biomarker-enriched advanced HCC populations. For this reason, we regard the guideline and regulatory status of Icaritin as clinical background only. It should not be taken as direct support for the TACE combined with Icaritin regimen evaluated in this retrospective cohort.

Several preclinical and translational studies have suggested that Icaritin may affect immune and metabolic pathways relevant to HCC, including FYN-related signaling and candidate metabolic targets (23, 24). These findings offer a plausible rationale for further study, especially in patients for whom conventional targeted or immune-based regimens are difficult to tolerate. Nevertheless, immune biomarkers, CBS status, and mechanistic endpoints were not assessed in this cohort. The present study therefore cannot determine whether the observed survival association was mediated through these pathways.

In a phase I trial by Fan et al., no grade 3 or higher drug-related adverse events occurred (3). In our cohort TACE with Icaritin did not significantly increase grade 3–4 liver-function-related adverse events or serious adverse events compared with TACE alone. It should be noted that multiple real-world studies such as the ELEVATOR cohort and the Korean LINK database have confirmed that TKIs like sorafenib/lenvatinib have high incidences of grade 3/4 adverse events (hand-foot syndrome, hypertension, proteinuria, etc.), particularly pronounced in Child-Pugh B patients, often requiring dose reduction or interruption (25, 26). Moreover, a study by Choi et al. showed that in Child-Pugh B patients, ICIs not only have significantly lower objective response rates (ORR) compared to grade A patients (e.g., 2.8% vs. 15.9%) but also carry a very high risk of immune-related adverse events (irAEs) requiring monitoring and management (27). However, this was not a head-to-head comparison between Icaritin and other systemic agents. It also cannot establish that Icaritin is preferable for patients with Child-Pugh grade B liver function. Given the small size and limited power of that subgroup, the results in Child-Pugh grade B patients should be viewed as exploratory. Prospective studies with biomarker collection, standardized TACE protocols, and prespecified liver-function strata are needed before this approach can be translated into a treatment recommendation.

This study has several limitations. Although PSM and IPTW were applied to reduce baseline imbalance, the retrospective design does not allow causal inference, and residual confounding cannot be fully excluded. Some clinically relevant factors, including prior treatment history, post-progression therapies, detailed TACE technique, and Icaritin dose interruption or modification, were not uniformly available across centers and therefore could not be completely adjusted. In addition, the Child-Pugh B subgroup was relatively small, so findings in this population should be interpreted as exploratory rather than definitive. CBS biomarker status was also unavailable, limiting assessment of whether the observed benefit differed according to the biomarker context established in prior Icaritin studies. Furthermore, due to practical constraints of real-world clinical practice, prospective peripheral blood biomarker testing was not performed for all patients. Finally, although mRECIST-based radiologic assessment is a validated method for evaluating tumor response in HCC and was used to define both response endpoints and imaging-confirmed PFS in this study, categorical response measures such as ORR and DCR do not capture response duration, timing of progression, liver-function trajectory, treatment tolerability, or subsequent treatment course. Therefore, the discrepancy between similar ORR/DCR and improved survival should be interpreted cautiously, rather than viewed as proof of a direct radiologic response advantage. Prospective studies with standardized treatment protocols, biomarker assessment, and detailed treatment-sequence data are warranted to validate these findings.

In conclusion, TACE with Icaritin was associated with improved survival in this retrospective cohort, particularly among patients with preserved liver function. The findings in patients with Child–Pugh grade B liver function should be interpreted with caution, as the observed PFS benefit in this subgroup was exploratory due to the limited sample size and insufficient statistical power, and therefore requires further validation. Future prospective, multicenter randomized controlled trials are warranted to further validate the efficacy and safety of this combination strategy and to explore the optimal timing and dosage.

## Data Availability

The data analyzed in this study is subject to the following licenses/restrictions: The datasets are not publicly available due to patient privacy and ethical restrictions. They can be requested from the corresponding author upon reasonable request, subject to institutional ethical approval. Requests to access these datasets should be directed to 18468198374@163.com.
