# Supplementary material for: Efficacy analysis of icaritin combined with transarterial chemoembolization in hepatocellular carcinoma stratified by different child-pugh classes
Source: Front Immunol. 2026 Jul 30;17:1876151. doi: 10.3389/fimmu.2026.1876151 (PMC13469161; doi:10.3389/fimmu.2026.1876151)
Supplement: Supplementary file 1 [file DataSheet1.docx]

**Figure Legend**

**Fig S1. Love plot with propensity score matching.**

**Fig S2. Subgroup analysis of overall survival after propensity score matching. Forest plot showing the association between TACE plus Icaritin and overall survival compared with TACE alone across clinically relevant subgroups in the propensity score-matched cohort. Hazard ratios (HRs) and 95% confidence intervals (CIs) were estimated using Cox proportional hazards models within each subgroup. An HR <1 indicates a survival benefit favoring TACE plus Icaritin. Interaction P values were calculated using treatment-by-subgroup interaction terms. The vertical dashed line represents HR = 1. N and event numbers are shown for each subgroup. OS, overall survival; TACE, transarterial chemoembolization; ECOG PS, Eastern Cooperative Oncology Group performance status; ALBI, albumin-bilirubin; AFP, alpha-fetoprotein.**

**Fig S3. Subgroup analysis of** **progression-free survival after propensity score matching.** **Forest plot showing the association between TACE plus Icaritin and progression-free survival compared with TACE alone across clinically relevant subgroups in the propensity score-matched cohort. Hazard ratios (HRs) and 95% confidence intervals (CIs) were estimated using Cox proportional hazards models within each subgroup. An HR <1 indicates a survival benefit favoring TACE plus Icaritin. Interaction P values were calculated using treatment-by-subgroup interaction terms. The vertical dashed line represents HR = 1. N and event numbers are shown for each subgroup. PFS, progression-free survival; TACE, transarterial chemoembolization; ECOG PS, Eastern Cooperative Oncology Group performance status; ALBI, albumin-bilirubin; AFP, alpha-fetoprotein.**

**Fig S4. Love plot with inverse probability of treatment weighting.**

**Fig S5. Landmark analysis of the Overall survival of the two groups with the cut-off values of the 4th, 6th, and 8th months under different Child-Pugh grades after IPTW.** (A). Child-Pugh grade A, the cut-off value is the 4th month. (B). Child-Pugh grade A, the cut-off value is the 6th month. (C). Child-Pugh grade A, the cut-off value is the 8th month. (D). Child-Pugh grade B, the cut-off value is the 4th month. (E). Child-Pugh grade B, the cut-off value is the 6th month. (F). Child-Pugh grade B, the cut-off value is the 8th month. IPTW, inverse probability of treatment weighting.

**Fig S6. Landmark analysis of the Progression-free survival of the two groups with the cut-off values of the 2nd, 4th, and 6th months under different Child-Pugh grades after IPTW.** (A). Child-Pugh grade A, the cut-off value is the 2nd month. (B). Child-Pugh grade A, the cut-off value is the 4th month. (C). Child-Pugh grade A, the cut-off value is the 6th month. (D). Child-Pugh grade B, the cut-off value is the 2nd month. (E). Child-Pugh grade B, the cut-off value is the 4th month. (F). Child-Pugh grade B, the cut-off value is the 6th month. IPTW, inverse probability of treatment weighting.

**Fig S7. Kaplan-Meier curves of Overall survival and Progression-free survival in two groups.** (A). Overall survival periods of the two groups under Overall Cohort. (B). Overall survival periods of the two groups under Child-Pugh grade A. (C). Overall survival periods of the two groups under Child-Pugh grade B. (D). Progression-free survival periods of the two groups under Overall Cohort. (E). Progression-free survival in both groups under Child-Pugh grade A. (F). Progression-free survival in both groups under Child-Pugh grade B.

**Fig S8. Fine-Gray analysis under different Child-Pugh grades.** (A). The overall cumulative incidence of PFS under Child-Pugh grade A. (B). Comparison of cumulative incidence rates between the two groups under Child-Pugh grade A. (C). The overall cumulative incidence of PFS under Child-Pugh grade B. (D). Comparison of cumulative incidence rates between the two groups under Child-Pugh grade B.

**Table S1. Distribution of BCLC stage before and after propensity score matching**

| Cohort | BCLC stage | TACE alone | TACE with Icaritin | P |
| --- | --- | --- | --- | --- |
| Before PSM | Stage B | 53 (34.0) | 64 (43.2) | 0.123 |
| Before PSM | Stage C | 103 (66.0) | 84 (56.8) | 0.123 |
| After PSM | Stage B | 44 (35.2) | 55 (44.0) | 0.196 |
| After PSM | Stage C | 81 (64.8) | 70 (56.0) | 0.196 |

Abbreviations: BCLC, Barcelona Clinic Liver Cancer; PSM, propensity score matching; TACE, transarterial chemoembolization. P values were calculated using the chi-square test.

| **Table S2. Baseline Covariates Before and After Matching** | | | | | | | | |
| --- | --- | --- | --- | --- | --- | --- | --- | --- |
| Variables | Level | Before Matching | | |  | After Matching | | |
|  |  | TACE alone | TACE with Icaritin | SMD |  | TACE alone | TACE with Icaritin | SMD |
| n |  | 156 | 148 |  |  | 125 | 125 |  |
| Sex (%) | Male | 141 (90.4) | 132 (89.2) | -0.038 |  | 113 (90.4) | 111 (88.8) | -0.052 |
|  | Female | 15 (9.6) | 16 (10.8) | 0.038 |  | 12 (9.6) | 14 (11.2) | 0.052 |
| Age (%) | ＜60 | 94 (60.3) | 91 (61.5) | 0.025 |  | 74 (59.2) | 77 (61.6) | 0.049 |
|  | ≥ 60 | 62 (39.7) | 57 (38.5) | -0.025 |  | 51 (40.8) | 48 (38.4) | -0.049 |
| ECOG (%) ^a^ | 0 | 104 (66.7) | 101 (68.2) | 0.034 |  | 84 (67.2) | 85 (68.0) | 0.017 |
|  | 1 | 50 (32.1) | 45 (30.4) | -0.036 |  | 39 (31.2) | 38 (30.4) | -0.017 |
|  | 2 | 2 (1.3) | 2 (1.4) | 0.006 |  | 2 (1.6) | 2 (1.6) | 0.000 |
| Targeted therapy (%) | None | 49 (31.4) | 46 (31.1) | -0.007 |  | 38 (30.4) | 36 (28.8) | -0.035 |
|  | Lenvatinib | 59 (37.8) | 60 (40.5) | 0.055 |  | 51 (40.8) | 54 (43.2) | 0.049 |
|  | Donafenib | 38 (24.4) | 36 (24.3) | -0.001 |  | 31 (24.8) | 29 (23.2) | -0.037 |
|  | Regorafenib | 10 (6.4) | 6 (4.1) | -0.119 |  | 5 (4.0) | 6 (4.8) | 0.041 |
| Extrahepatic metastases (%) | No | 142 (91.0) | 136 (91.9) | 0.032 |  | 115 (92.0) | 117 (93.6) | 0.059 |
|  | Yes | 14 (9.0) | 12 (8.1) | -0.032 |  | 10 (8.0) | 8 (6.4) | -0.059 |
| Viral infection (%) | No | 2 (1.3) | 8 (5.4) | 0.182 |  | 2 (1.6) | 2 (1.6) | 0.000 |
|  | Hepatitis B | 142 (91.0) | 133 (89.9) | -0.038 |  | 120 (96.0) | 117 (93.6) | -0.080 |
|  | Hepatitis C | 9 (5.8) | 6 (4.1) | -0.087 |  | 3 (2.4) | 5 (4.0) | 0.081 |
|  | Other | 3 (1.9) | 1 (0.7) | -0.152 |  | 0 (0.0) | 1 (0.8) | 0.098 |
| Portal vein tumor thrombus (%) | None | 73 (46.8) | 92 (62.2) | 0.317 |  | 68 (54.4) | 70 (56.0) | 0.033 |
|  | Type Ⅰ | 24 (15.4) | 20 (13.5) | -0.055 |  | 20 (16.0) | 20 (16.0) | 0.000 |
|  | Type Ⅱ | 49 (31.4) | 29 (19.6) | -0.298 |  | 31 (24.8) | 29 (23.2) | -0.040 |
|  | Type Ⅲ | 9 (5.8) | 6 (4.1) | -0.087 |  | 5 (4.0) | 5 (4.0) | 0.000 |
|  | Type Ⅳ | 1 (0.6) | 1 (0.7) | 0.004 |  | 1 (0.8) | 1 (0.8) | 0.000 |
| AFP≥400 ng/mL (%) | No | 106 (67.9) | 93 (62.8) | -0.106 |  | 84 (67.2) | 79 (63.2) | -0.083 |
|  | Yes | 50 (32.1) | 55 (37.2) | 0.106 |  | 41 (32.8) | 46 (36.8) | 0.083 |
| Number of lesions (%) | ≤ 3 | 121 (77.6) | 103 (69.6) | -0.173 |  | 95 (76.0) | 90 (72.0) | -0.087 |
|  | ＞3 | 35 (22.4) | 45 (30.4) | 0.173 |  | 30 (24.0) | 35 (28.0) | 0.087 |
| Maximum diameter of lesion (%) | ＜5 cm | 61 (39.1) | 47 (31.8) | -0.158 |  | 51 (40.8) | 43 (34.4) | -0.137 |
|  | ≥ 5 cm | 95 (60.9) | 101 (68.2) | 0.158 |  | 74 (59.2) | 82 (65.6) | 0.137 |
| Vascular invasion (%) | No | 3 (1.9) | 15 (10.1) | 0.272 |  | 3 (2.4) | 5 (4.0) | 0.053 |
|  | Yes | 153 (98.1) | 133 (89.9) | -0.272 |  | 122 (97.6) | 120 (96.0) | -0.053 |
| Abbreviations: TACE, transarterial chemoembolization; SMD, Standardized Mean Difference ECOG, Eastern Cooperative Oncology Group; AFP, alpha-Fetoprotein. ^a^ ECOG score of 0 indicates that patient is fully active and able to carry on all pre-disease activities without restriction, and 1 indicates that patient is restricted in physically strenuous activity but is ambulatory and able to carry out work of a light nature, including self-care. | | | | | | | | |

| **Table S3. Median Overall Survival and Progression-free Survival and 1-year Survival Rate** | | | | | | | | | | | | | |
| --- | --- | --- | --- | --- | --- | --- | --- | --- | --- | --- | --- | --- | --- |
| Grade | Group | Before PSM | | | | | | After PSM | | | | | |
|  |  | OS | | | PFS | | | OS | | | PFS | | |
|  |  | Median | 1-year survival rate | P^a^ | Median | 1-year survival rate | P^a^ | Median | 1-year survival rate | P^a^ | Median | 1-year survival rate | P^a^ |
| A | TACE alone | 13.5 | 56% | 0.026 | 7.6 | 15% | 0.018 | 15.7 | 64% | ＜0.001 | 8.1 | 19% | 0.007 |
|  | TACE with Icaritin | 16.9 | 67% |  | 8.4 | 26% |  | 28.8 | 80% |  | 9.7 | 34% |  |
| B | TACE alone | 13.0 | 52% | 0.030 | 6.6 | 0 | 0.109 | 11.3 | 46% | 0.015 | 5.2 | 4.0% | 0.017 |
|  | TACE with Icaritin | 14.4 | 58% |  | 7.0 | 27% |  | 18.7 | 66% |  | 6.0 | 44% |  |
| Abbreviations: PSM, propensity score matching; OS, overall survival; PFS, progression-free survival; TACE, transarterial chemoembolization. All survival times are expressed in months. One-year survival rates are presented as percentages (%).  ^a^ P values were calculated using the log-rank test. | | | | | | | | | | | | | |

**Table S4. BCLC-stratified and BCLC-adjusted Cox analyses after propensity score matching**

| Analysis | Endpoint | n/events | Median, TACE alone | Median, TACE with Icaritin | HR (95% CI) | P |
| --- | --- | --- | --- | --- | --- | --- |
| BCLC stage B: TACE with Icaritin vs TACE alone | OS | 99/40 | 21.00 | NR | 0.30 (0.15-0.59) | <0.001 |
| BCLC stage C: TACE with Icaritin vs TACE alone | OS | 151/116 | 12.50 | 16.00 | 0.53 (0.36-0.78) | 0.001 |
| BCLC-adjusted model: TACE with Icaritin vs TACE alone | OS | 250/156 | - | - | 0.50 (0.35-0.70) | <0.001 |
| BCLC-adjusted model: BCLC stage C vs B | OS | 250/156 | - | - | 3.46 (2.30-5.20) | <0.001 |
| BCLC stage B: TACE with Icaritin vs TACE alone | PFS | 99/84 | 8.80 | 11.83 | 0.72 (0.47-1.11) | 0.133 |
| BCLC stage C: TACE with Icaritin vs TACE alone | PFS | 151/131 | 6.10 | 8.47 | 0.58 (0.41-0.82) | 0.002 |
| BCLC-adjusted model: TACE with Icaritin vs TACE alone | PFS | 250/215 | - | - | 0.62 (0.47-0.81) | <0.001 |
| BCLC-adjusted model: BCLC stage C vs B | PFS | 250/215 | - | - | 1.47 (1.09-1.99) | 0.012 |

Abbreviations: BCLC, Barcelona Clinic Liver Cancer; CI, confidence interval; HR, hazard ratio; NR, not reached; OS, overall survival; PFS, progression-free survival; PSM, propensity score matching; TACE, transarterial chemoembolization. HR < 1 favors TACE with Icaritin for treatment effect. BCLC stage C was defined as ECOG performance status 1-2 and/or the presence of portal vein tumor thrombus or extrahepatic metastasis; patients without these features were classified as BCLC stage B. Adjusted Cox models included treatment group, BCLC stage, age, sex, Child-Pugh class, AFP >=400 ng/mL, number of lesions, maximum tumor diameter, and targeted therapy.

**Table S5. Complete response-adjusted Cox sensitivity analysis for overall survival after propensity score matching**

| Variable | Category | Univariable HR (95% CI) | P | Multivariable HR (95% CI) | P |
| --- | --- | --- | --- | --- | --- |
| Treatment group | TACE alone | Reference | Reference | Reference | Reference |
|  | TACE with Icaritin | 0.44 (0.31-0.61) | <0.001 | 0.49 (0.35-0.70) | <0.001 |
| Best mRECIST response | CR | Reference | Reference | Reference | Reference |
|  | PR | 1.46 (0.74-2.90) | 0.272 | 6.57 (2.81-15.39) | <0.001 |
|  | PD | 1.14 (0.75-1.71) | 0.542 | 2.38 (1.45-3.90) | <0.001 |
|  | SD | 0.96 (0.62-1.49) | 0.863 | 1.86 (1.10-3.13) | 0.021 |
| Additional targeted therapy during follow-up | None | Reference | Reference | Reference | Reference |
|  | Lenvatinib | 1.13 (0.76-1.68) | 0.547 | 1.07 (0.70-1.64) | 0.748 |
|  | Donafenib | 1.23 (0.79-1.93) | 0.362 | 1.69 (1.04-2.75) | 0.033 |
|  | Regorafenib | 2.12 (1.10-4.08) | 0.024 | 1.59 (0.78-3.27) | 0.205 |
| Age | <60 years | Reference | Reference | Reference | Reference |
|  | >=60 years | 1.13 (0.83-1.55) | 0.440 | 1.32 (0.94-1.86) | 0.108 |
| Sex | Male | Reference | Reference | Reference | Reference |
|  | Female | 0.49 (0.29-0.84) | 0.010 | 0.52 (0.29-0.93) | 0.028 |
| ECOG performance status | 0 | Reference | Reference | Reference | Reference |
|  | 1 | 2.93 (2.10-4.07) | <0.001 | 2.11 (1.47-3.04) | <0.001 |
|  | 2 | 8.71 (2.68-28.25) | <0.001 | 6.70 (1.91-23.49) | 0.003 |
| Child-Pugh class | Grade A | Reference | Reference | Reference | Reference |
|  | Grade B | 2.10 (1.46-3.02) | <0.001 | 1.39 (0.92-2.09) | 0.114 |
| Portal vein tumor thrombus | No | Reference | Reference | Reference | Reference |
|  | Yes | 3.97 (2.82-5.59) | <0.001 | 2.88 (1.96-4.23) | <0.001 |
| Extrahepatic metastasis | No | Reference | Reference | Reference | Reference |
|  | Yes | 2.54 (1.43-4.50) | 0.001 | 2.09 (1.01-4.31) | 0.047 |
| AFP | <400 ng/mL | Reference | Reference | Reference | Reference |
|  | >=400 ng/mL | 1.17 (0.86-1.61) | 0.322 | 1.05 (0.73-1.49) | 0.807 |
| Number of lesions | <=3 | Reference | Reference | Reference | Reference |
|  | >3 | 2.71 (1.75-4.20) | <0.001 | 2.33 (1.41-3.84) | <0.001 |
| Maximum tumor diameter | <5 cm | Reference | Reference | Reference | Reference |
|  | >=5 cm | 1.48 (1.02-2.13) | 0.037 | 1.19 (0.78-1.80) | 0.418 |
| Vascular invasion | No | Reference | Reference | Reference | Reference |
|  | Yes | 2.81 (1.24-6.35) | 0.013 | 2.23 (0.95-5.23) | 0.065 |

Abbreviations: AFP, alpha-fetoprotein; CI, confidence interval; CR, complete response; ECOG, Eastern Cooperative Oncology Group; HR, hazard ratio; mRECIST, modified Response Evaluation Criteria in Solid Tumors; OS, overall survival; PD, progressive disease; PFS, progression-free survival; PR, partial response; PSM, propensity score matching; SD, stable disease; TACE, transarterial chemoembolization. The multivariable model included treatment group, best mRECIST response category, additional targeted therapy during follow-up, age, sex, ECOG performance status, Child-Pugh class, portal vein tumor thrombus, extrahepatic metastasis, AFP >=400 ng/mL, number of lesions, maximum tumor diameter, and vascular invasion. Best mRECIST response is a post-baseline variable; therefore, these analyses are exploratory sensitivity analyses and should not be interpreted as causal mediation analyses.

**Table S6. Complete response-adjusted Cox sensitivity analysis for progression-free survival after propensity score matching**

| Variable | Category | Univariable HR (95% CI) | Univariable P | Multivariable HR (95% CI) | Multivariable P |
| --- | --- | --- | --- | --- | --- |
| Treatment group | TACE alone | Reference | Reference | Reference | Reference |
|  | TACE with Icaritin | 0.61 (0.47-0.80) | <0.001 | 0.60 (0.45-0.81) | <0.001 |
| Best mRECIST response | CR | Reference | Reference | Reference | Reference |
|  | PR | 0.88 (0.48-1.61) | 0.678 | 1.74 (0.85-3.55) | 0.130 |
|  | PD | 1.36 (0.95-1.96) | 0.094 | 2.14 (1.40-3.28) | <0.001 |
|  | SD | 1.26 (0.86-1.85) | 0.227 | 2.05 (1.31-3.18) | 0.002 |
| Additional targeted therapy during follow-up | None | Reference | Reference | Reference | Reference |
|  | Lenvatinib | 1.14 (0.82-1.59) | 0.435 | 1.22 (0.86-1.73) | 0.269 |
|  | Donafenib | 1.33 (0.91-1.93) | 0.140 | 1.55 (1.04-2.31) | 0.032 |
|  | Regorafenib | 1.59 (0.85-2.98) | 0.147 | 1.82 (0.94-3.52) | 0.075 |
| Age | <60 years | Reference | Reference | Reference | Reference |
|  | >=60 years | 0.99 (0.76-1.29) | 0.936 | 0.89 (0.67-1.18) | 0.410 |
| Sex | Male | Reference | Reference | Reference | Reference |
|  | Female | 0.70 (0.47-1.06) | 0.093 | 0.82 (0.53-1.27) | 0.372 |
| ECOG performance status | 0 | Reference | Reference | Reference | Reference |
|  | 1 | 1.51 (1.12-2.03) | 0.007 | 1.39 (1.00-1.92) | 0.047 |
|  | 2 | 3.18 (1.01-10.04) | 0.049 | 3.84 (1.15-12.80) | 0.028 |
| Child-Pugh class | Grade A | Reference | Reference | Reference | Reference |
|  | Grade B | 1.40 (1.00-1.96) | 0.052 | 1.17 (0.80-1.69) | 0.423 |
| Portal vein tumor thrombus | No | Reference | Reference | Reference | Reference |
|  | Yes | 1.80 (1.37-2.36) | <0.001 | 1.58 (1.16-2.14) | 0.004 |
| Extrahepatic metastasis | No | Reference | Reference | Reference | Reference |
|  | Yes | 1.63 (0.96-2.78) | 0.071 | 2.02 (1.09-3.75) | 0.026 |
| AFP | <400 ng/mL | Reference | Reference | Reference | Reference |
|  | >=400 ng/mL | 0.95 (0.72-1.25) | 0.716 | 0.94 (0.70-1.27) | 0.704 |
| Number of lesions | <=3 | Reference | Reference | Reference | Reference |
|  | >3 | 1.51 (1.10-2.07) | 0.012 | 1.43 (0.99-2.05) | 0.056 |
| Maximum tumor diameter | <5 cm | Reference | Reference | Reference | Reference |
|  | >=5 cm | 1.30 (0.97-1.75) | 0.078 | 1.29 (0.93-1.81) | 0.132 |
| Vascular invasion | No | Reference | Reference | Reference | Reference |
|  | Yes | 0.98 (0.63-1.55) | 0.947 | 0.74 (0.46-1.20) | 0.226 |

Abbreviations: AFP, alpha-fetoprotein; CI, confidence interval; CR, complete response; ECOG, Eastern Cooperative Oncology Group; HR, hazard ratio; mRECIST, modified Response Evaluation Criteria in Solid Tumors; OS, overall survival; PD, progressive disease; PFS, progression-free survival; PR, partial response; PSM, propensity score matching; SD, stable disease; TACE, transarterial chemoembolization. The multivariable model included treatment group, best mRECIST response category, additional targeted therapy during follow-up, age, sex, ECOG performance status, Child-Pugh class, portal vein tumor thrombus, extrahepatic metastasis, AFP >=400 ng/mL, number of lesions, maximum tumor diameter, and vascular invasion. Best mRECIST response is a post-baseline variable; therefore, these analyses are exploratory sensitivity analyses and should not be interpreted as causal mediation analyses.

| Class | Group | Raw n/N | Weighted n/N | Median OS (95%CI) | 12-month OS | 24-month OS | Weighted HR | Weighted P |
| --- | --- | --- | --- | --- | --- | --- | --- | --- |
| A | TACE alone | 102/124 | 189.8/243.1 | 15.0 (13.0-19.1) | 64.5% | 22.7% | Reference |  |
|  | TACE with Icaritin | 49/123 | 103.4/250.4 | 25.4 (20.6-NE) | 83.7% | 34.4% | 0.46 (0.33-0.65) | <0.001 |
| B | TACE alone | 31/32 | 54.1/57.8 | 12.8 (8.2-15.5) | 56.4% | 4.7% | Reference |  |
|  | TACE with Icaritin | 16/25 | 30.5/52.0 | 15.0 (14.4-NE) | 70.0% | 21.1% | 0.48 (0.25-0.94) | 0.031 |

Table S7. IPTW-weighted overall survival according to Child-Pugh class.

NE, not estimable. Hazard ratios were estimated using IPTW-weighted Cox proportional hazards models with robust standard errors. HR <1 favors TACE plus Icaritin.

| Table S8. Adverse Events | | | | | | |
| --- | --- | --- | --- | --- | --- | --- |
|  | Any grade, n (%) | | | Grade 3-4, n(%) | | |
| Event | TACE with Icaritin (n = 125) | TACE alone (n = 125) | P | TACE with Icaritin (n = 125) | TACE alone (n = 125) | P |
| Total bilirubin increased | 108 (86.3) | 105 (84.1) | 0.593 | 23 (18.4) | 18 (14.4) | 0.393 |
| GGT increased | 96 (76.5) | 102 (81.6) | 0.350 | 3 (2.4) | 2 (1.6) | >0.999 |
| ALT increased | 116 (92.8) | 114 (91.2) | 0.641 | 16 (12.8) | 17 (13.6) | 0.852 |
| AST increased | 107 (85.7) | 102 (81.6) | 0.393 | 14 (11.2) | 12 (9.6) | 0.679 |
| Diarrhea | 36 (28.8) | 31 (24.8) | 0.475 | 4 (3.2) | 3 (2.4) | >0.999 |
| PT prolonged | 49 (39.29) | 43 (34.4) | 0.431 | 2 (1.6) | 1 (0.8) | >0.999 |
| Hypoalbuminemia | 70 (56.0) | 61 (48.8) | 0.254 | 4 (3.2) | 3 (2.4) | >0.999 |
| Hand-footskinreaction | 13 (10.4) | 17 (13.6) | 0.436 | 2 (1.6) | 3 (2.4) | >0.999 |
| Ascites worsening | 30 (24.0) | 27 (21.6) | 0.651 | 3 (2.4) | 2 (1.6) | >0.999 |
| Abdominal pain | 108 (86.4) | 111 (88.8) | 0.565 | 28 (22.4) | 37 (29.6) | 0.194 |
| Hepatic encephalopathy | 12 (9.6) | 10 (8.0) | 0.655 | 3 (2.4) | 2 (1.6) | >0.999 |
| Gastrointestinal bleeding | 12 (9.6) | 9 (7.2) | 0.494 | 4 (3.2) | 2 (1.6) | 0.684 |
| Nausea | 102 (81.6) | 95 (76.0) | 0.279 | 22 (17.6) | 19 (15.2) | 0.608 |
| Thrombocytopenia | 82 (65.6) | 99 (79.2) | 0.016 | 10 (8.0) | 11 (8.8) | 0.820 |
| Albuminuria | 28 (22.4) | 21 (16.8) | 0.265 | 2 (1.6) | 1 (0.8) | >0.999 |
| Pruritus | 11 (8.8) | 12 (9.6) | 0.827 | 2 (1.6) | 3 (2.4) | >0.999 |
| Fever | 77 (61.6) | 82 (65.6) | 0.511 | 20 (16.0) | 17 (13.6) | 0.593 |
| Weight decreased | 47 (37.6) | 40 (32.0) | 0.353 | 3 (2.4) | 2 (1.6) | >0.999 |
| Anemia | 32 (25.6) | 30 (24.0) | 0.770 | 4 (3.2) | 6 (4.8) | 0.519 |
| Hypertension | 20 (16.0) | 15 (12.0) | 0.362 | 5 (4.0) | 4 (3.2) | >0.999 |
| WBC count decreased | 28 (22.4) | 25 (20.0) | 0.642 | 2 (1.6) | 1 (0.8) | >0.999 |
| Electrolyte imbalance | 28 (22.4) | 23 (18.4) | 0.433 | 5 (4.0) | 3 (2.4) | 0.722 |

Abbreviations: Icaritin-TACE, transarterial chemoembolization plus Icaritin; TACE, transarterial chemoembolization; GGT, gamma-glutamyl transferase; ALT, alanine aminotransferase; AST, aspartate aminotransferase; WBC, white blood cell; PT, prothrombin time. P values calculated by Chi-square test (or Fisher’s exact test if expected cell count <5).
